# A Pathogenesis Related Protein, VpPR-10.1, from *Vitis pseudoreticulata*: An Insight of Its Mode of Antifungal Activity

**DOI:** 10.1371/journal.pone.0095102

**Published:** 2014-04-23

**Authors:** Teng-Fei Xu, Xiao-Chen Zhao, Yun-Tong Jiao, Jin-Yu Wei, Lan Wang, Yan Xu

**Affiliations:** 1 State Key Laboratory of Crop Stress Biology in Arid Areas (Northwest A&F University), Yangling, Shaanxi, China; 2 College of Horticulture, Northwest A&F University, Yangling, Shaanxi, China; 3 Key Laboratory of Horticultural Plant Biology and Germplasm Innovation in Northwest China, Ministry of Agriculture, Yangling, Shaanxi, China; 4 Shandong Seed Group Co., LTD., Jinan, Shandong, China; Key Laboratory of Horticultural Plant Biology (MOE), China

## Abstract

Previously, *VpPR-10.1* was isolated and characterized from a cDNA library of a fungus-resistant accession of Chinese wild grape (*Vitis pseudoreticulata*). We found that expression of *VpPR-10.1* is affected by the fungal pathogen *Erysiphe necator*. To investigate the biochemical basis of the nuclease activity of *VpPR-10.1* and its role in antifungal resistance, we generated recombinant VpPR-10.1 as well as site-directed mutations targeting three conserved amino acid residues among plant PR-10 s: Lys55, Glu149, and Tyr151. We showed that wild-type recombinant VpPR-10.1 exhibits both RNase and DNase activities. Mutant VpPR10.1-Y151H essentially retained all these activities. In contrast, VpPR10.1-K55N, where Lys55 in the P-loop region is mutated to Asn, and VpPR10.1-E149G, where Glu149 is mutated to Gly, lost their nuclease activity, indicating that both residues play a critical role in catalyzing RNA and DNA degradation. Furthermore, VpPR10.1 and VpPR10.1-Y151H inhibited the growth of the cultured fungal pathogen *Alternaria alternate*. Through transient expression in grapevine, we also demonstrated that VpPR10.1-K55N and VpPR10.1-E149G compromised resistance to *E. necator*. Finally, we further found that VpPR-10.1 can lead to programmed cell death and DNA degradation when incubated with tobacco BY-2 suspension cells. We show here that Lys55 and Glu149, but not Tyr151, are required for the RNase, DNase and antifungal activities of VpPR-10.1. The strong correlation between the level of VpPR-10.1 nuclease activity and its antifungal property indicates that the former is the biochemical basis for the latter. Taken together, our experiments revealed that VpPR-10.1 is critical in mediating fungal resistance in grape, potentially playing a dual role by degrading pathogen RNA and inducing programmed death of host cells.

## Introduction

Plants synthesize several kinds of defense proteins when they are exposed to pathogens or environmental stresses, including phytoalexins, lytic enzymes, proteinase inhibitors and low molecular weight proteins, defined as pathogenesis-related (PR) proteins [1], [2]. Plant PR proteins were first described in tobacco leaves infected by tobacco mosaic virus (TMV) [3]. They have since been identified in both monocot and dicot plant species. PR proteins do not usually accumulate in healthy plants, but are induced by pathogen infection or related stresses. Thus, they play various roles to improve the defensive capacity of plants [4].

PR proteins are grouped into 17 families, based on their sequence, structure and biological activities [5]. Most of them are extracellular proteins or intracellularly localized in the vacuole. In contrast, PR-10 proteins are present in the cytoplasm because they lack a signal peptide and constitute one of the most important PR families in response to fungal invasion [6]. Generally, PR-10 proteins are slightly acidic, with a molecular mass of 16–19 kDa [7]. They were first identified in cultured parsley cells after treatment with an elicitor [8]. To date, members of the PR-10 family have been reported in a variety of higher plant species of both monocots [9], [10] and dicots [7], [11]–[14]. Several PR-10 genes are expressed in different tissues and organs during plant growth and development [15], [16], such as the pollen grain [11], [17], flower organs [11], [18]–[21], fruit [22], [23], seeds [21], [24], vegetative organs of roots [25]–[28], stems [21], [29] and leaves [29], [30].

PR-10 proteins play important roles in plant defense in response to different conditions. The expression of PR-10 genes is induced by pathogens and related stresses. Pathogens triggering a PR-10 response include viruses [23], [31]–[33], bacteria [13], [14], [34] and fungi [12], [29], [32], [35]–[38]. The recombinant CaPR-10 protein from hot pepper (*Capsicum annuum*) inhibits the growth of the oomycete pathogen *P. capsici* [31]. Expression of the pea PR-10.1 gene in potato confers resistance to early dying disease [39]. Expression of PR-10 genes is also induced by other abiotic stresses, such as high salinity [40], drought [31], [41], dormancy [42], copper stress and other related oxidative stress [43], [44], ultraviolet radiation [32] and wounding [18], [29], [37], [45], [46]. Furthermore, plant hormones and defense-related signaling molecules modulate PR-10 expression, including jasmonic acid [38], [40], [47], [48], abscisic acid (ABA) [48] and salicylic acid [38]. Besides, as an important environmental factor, cold stress affects PR-10 expression in ‘Loring’ peach (*Prunus persica*) [49] and mulberry [16]. In winter, the accumulation has the highest level in the roots of sugar pine and western white pine [50]. These observations imply that PR-10 genes are important in the process of plant development and defense responses.

PR-10 proteins are reported to share sequence homology with ginseng ribonuclease. Several PR-10 proteins were tested *in vitro* and confirmed to have ribonuclease activity, including Bet v 1 from birch (*Betula verrucosa*) pollen [19], [51], LaPR-10 from lupine (*Lupinus albus*) roots [52] and PR-10c from birch (*Betula pendula*) [53]. Most PR-10 proteins comprise two domains. One is the phosphate-binding loop (P-loop; GXGGXG) that is highly conserved among nucleotide-binding proteins [54]; the other is the Bet v 1 motif, which is characteristic of proteins from the Bet v 1 superfamily [55]. The P-loop is believed to be involved in ATP or GTP binding and is critical for the RNase activity of SPE-16, a PR-10 protein from the seeds of *Pachyrrhizus erosus* [24]. Chadha and Das [56] reported that mutant protein AhPR-10-K54N (positioned in the P-loop motif) lost its ribonuclease and antifungal activities. Several amino acids in the Bet v 1 motif (E96, E148 and Y150) are highly conserved and implicated in the ribonuclease activity [55]. E147A and Y149A mutations of SPE-16 drastically decreased the ribonuclease activity [24]. Similarly, E148K and Y150F mutations to GaPR-10 abolished its RNase activity, while the E96K mutation decreased the activity to half [9]. A yeast tRNA-degradation test showed that phosphorylated CaPR-10 has higher RNase activity than the non-phosphorylated form [31], suggesting that phosphorylation modulates the RNase activity.

At present, in grapevine *Vitis vinifera*, 17 PR-10 related genes have been described, which share high sequence similarity and are clustered on chromosome 5. Expression of three of these genes, *VvPR-10.1*, *VvPR-10.2* and *VvPR-10.3*, was detected during somatic embryogenesis (SE) induction [57]. At the same time, they displayed different expression levels in response to pathogen inoculation and salt or herbicide stresses [34], [58], [59]. In a previous work, we cloned a *PR-10* gene (designated as *VpPR-10.1*) from a fungal-resistant accession of Chinese wild *V. pseudoreticulata*, which encoded a 159-amino-acid polypeptide with a predicted molecular mass of 17.46 kDa [61]. The putative VpPR-10.1 protein has maximum amino acid sequence homology (89% and 79%) with two PR-10 proteins from *V. vinifera* Ugni Blanc, respectively. VpPR-10.1 is also structurally related to *Betula pendula* pollen allergen Betv1 (52% similarity) [61]. We found that the expression of *VpPR-10.1* varied at different times after inoculation with *E. necator*.

The PR-10 RNase activity is suggested to protect plants during programmed cell death around infection sites or to act directly on the pathogens [55]. Moreover, in rice suspension-cultured cells treated with the PBZ1 protein, DNA fragmentation, a hallmark of programmed cell death, was also detected [62]. Thus, in addition to RNase activity, PR-10 proteins may possess DNase activity that is involved in plant cell death. Indeed, we showed previously that the recombinant PR-10 protein from *V. pseudoreticulata* exhibited DNase activity against host genomic DNA and RNase activity against yeast total RNA *in vitro* [63]. However, it is not clear whether the RNase and DNase activities are encoded by the same amino acids and how the conserved sites in the P-loop and the Bet v 1 motifs are involved.

Here, we further investigated the biochemical basis of the RNase/DNase activity of VpPR-10 and its antifungal property using *in vitro* and *in vivo* assays. Critical motifs and conserved sites for these activities were analyzed and their involvements in cell death were observed. These results provide a better understanding of the role of PR-10 in the response to *E. necator* infection and will aid in the use of the Chinese wild grapevine *V. pseudoreticulata* for breeding.

## Materials and Methods

### Plant materials

Chinese wild *V. pseudoreticulata* accession Baihe-35-1 plants were grown in 10 cm pots filled with a mixture of 60% vermiculite and 40% meadow soil, and cultured in growth chambers (16 h light/8 h dark at 25–26°C). *In vitro* cultivation of the susceptible *V. vinifera* ‘Carignane’, used for transient experiments, was performed as described by Guan *et al*. [64].

The *E. necator*-infected leaves were collected from field-grown *V. vinifera* cv. Cabernet Sauvignon plants in the Grape Repository of Northwest A & F University, Yangling, Shaanxi, China. Inoculation by *E. necator* was performed on Chinese wild *V. pseudoreticulata* ‘Baihe-35-1’ under field conditions described previously [65]. Leaves of the *Vitis* were inoculated with *E. necator* and harvested at 24, 48, 72, 96, and 120 h post-inoculation, respectively. The inoculated leaves were immediately covered with paper bags to prevent infection with other pathogens, frozen immediately in liquid nitrogen and stored at −80°C until further use.

The suspension of tobacco BY-2 cells (*Nicotiana tabacum* L. cv. Bright Yellow 2) [66] was cultured in Murashige & Skoog medium, supplemented with 30 g·L^−1^ sucrose, 1 mg·L^−1^ thiamine, 100 mg·L^−1^ myo-inositol, 256 mg·L^−1^ KH_2_PO_4_ and 0.2 mg·L^−1^ 2,4-Dichlorophenoxyacetic acid (2,4-D), with a final pH of 5.7, adjusted with 1 M KOH. The cells were maintained on a rotary shaker at 120 rpm at 25 °C in the dark and sub-cultured weekly by 1∶50 dilution with fresh medium [67].

### PR10.1 gene cloning and PCR amplification

Total RNA was isolated from *V. pseudoreticulata* leaf samples after 0, 24, 48, 72, 96, 120, and 144 h of inoculation with *E. necator* by the LiCl precipitation method [68]. First-strand cDNA was prepared from 5 µg of the DNase-treated total mRNA in a 20 µL final volume using the PrimeScript reverse transcriptase kit ((Fermentas, Burlington, Canada)). The resulting cDNA served as the template for PCR amplification of *VpPR-10.1* (*GenBank no. DQ336289*).

PCR amplifications were performed using the forward primer Wild-F (5′-GGGGGATCCATGGGTGTTTTCACTTACGAG-3′) and reverse primer Wild-R (5′-GGGCTCGAGTTAATAGGCATCAGGGTGTGC 3′). Three substitution mutants (K55N, E149G, and Y151H) were constructed by site-directed mutagenesis using overlap extension PCR [69] with the following primer sets, K55N-F (5′-GGAACCATCAACAAGATTCAC-3′) and K55N-R (5′-GTGAATCTTGTTGATGGTTCC-3′); E149G-F (5′-ATGGGTGTTTTCACTTACGAG-3′) and E149G-R (5′- TTAATAGGCATCAGGGTGTGCAATGATGTAGGCTCCAAT-3′); Y151H-F (5′-ATGGGTGTTTTCACTTACGAG-3′) and Y151H-R (5′-TTAATAGGCATCAGGGTGTGCAATGATGTGGGC-3′). A *BamH* I restriction enzyme site (underlined sequences) was introduced at the 5′ end of the forward primer and an *Xho*I site (underlined sequence) was added at the 3′ end of the reverse primer. PCR reactions were carried out at an annealing temperature of 56 °C for 35 cycles. After ligating into vector pGEM-T Easy vector (Promega, Madison, WI, USA), DNA sequencing was used to confirm the amplicons. Similarity searches were conducted at the NCBI GenBank database (NCBI, http://www. ncbi.nlm.nih.gov/). Amino acid sequences were aligned using DNAMAN5.2 software (Lynnon Biosoft Corp.). Prediction of signal peptides was conducted on the SignalP 4.0 Server (http://www.cbs.dtu.dk/services/SignalP/).

### Expression and purification of recombinant proteins

*VpPR-10.1* and its mutated coding regions were digested with BamHI and XhoI, and sub-cloned into the expression vector pGEX-4T-1 to create an in-frame fusion with a GST affinity tag at the N-terminal end. The pGEX-4T-1 vectors containing wild-type *VpPR-10.1* and its mutants were transformed into *E. coli* BL21 (DE3) strain and grown in LB with 100 mg.mL^−1^ of ampicillin at 37 °C to an absorbance of 0.5–1.0 at 600 nm. Over-expression of the cloned genes was induced with 1 mM IPTG at 30 °C for 4 h. The expression and purification of recombinant proteins were performed according to the methods described by Xu *et al.* [61]. The bacterial cells were pelleted after incubation and suspended in BacReady-Protein Extraction Solution (Haigen, China). Fusion proteins were purified with Glutathione-Sepharose 4B resin (Pharmacia, Sweden) by affinity chromatography. The pGEX-4T-1 empty vector in BL21 (DE3) was used as a control. Considering the likely impact of GST tag on tertiary structure of target protein, GST tag was removed to avoid its effect on the function of target protein. To remove the GST tag, the fusion protein was treated using the Thrombin Cleavage Capture Kit (Novagen, Madison, WI, USA).

### RNase and DNase activities assays of recombinant proteins

To determine the RNase activity of the purified recombinant VpPR-10.1 and its mutants, RNase activity assays were performed according to the method described by Yan *et al.* [70] with some modifications. 200 µg of yeast tRNA and 100 µg of wild *V. pseudoreticulata* accession ‘Baihe-35-1’ total RNA were incubated with 100 µg of purified proteins (without GST) in 400 µL of 100 mM MES (2-(N-morpholino) ethanesulfonic acid, pH 7.0) at 37 °C for 30 min. The reactions were terminated using 500 µL CHCl_3_. The samples were centrifuged at 4 °C for 15 min at 12,000 rpm after leaving for 10 min at 4 °C. The supernatant was separated by electrophoresis through 1.0% agarose gels containing 0.75 µg·mL^−1^ ethidium bromide. RNase H was used as the positive control. Boiled wild-type recombinant VpPR-10.1 and boiled mutant proteins were used as negative controls. 100 U RNasin was added to the reactions (except for the sample with RNase H) to avoid contamination from foreign RNases.

DNase activity was analyzed using 10 µg of the purified protein. The enzyme was incubated with 4 µg of purified genomic DNA of wild *V. pseudoreticulata* accession ‘Baihe-35-1’ in a total volume of 50 µL, in the presence of 10 mM Tris-Cl pH 7.0 and 2.5 mM MgCl_2_, at 37 °C for 60 min. The reaction was terminated by adding 500 µL of CHCl_3_ to the mixture, which was then stored at 4 °C for 10 min, before being centrifuged at 12 000 rpm for 15 min. 10 µL of the supernatant was separated on 1.0% agarose gels and detected by electrophoresis under UV light.

### *In vitro* antifungal activity assays

The *in vitro* antifungal activities of VpPR-10.1 and its mutants were assayed by the spore growth inhibition method with modifications described by Chadha *et al*. [56] and Xu *et al*. [61]. Fungal pathogen *A. alternata* was used to check the antifungal activity of VpPR-10.1 and its mutants. The fungus had been pre-germinated on potato dextrose broth agar (PDB) plates at room temperature. The fungus was removed and suspended in 5 mL of sucrose solution (10% w/v). The fungal suspension was filtered through two layers of gauze to separate the sporangia. The concentration of sporangia was determined using a hemocytometer and adjusted to 1×10^5^ sporangia/ml. PDA agar plates with 10 µL of protein samples at different concentrations were used to grow spores containing the same number of sporangia (20 µL, 1×10^5^ sporangia/mL), which were then dried and cultured at room temperature. PDB agar plates with boiled recombinant VpPR-10.1 and mutant proteins were used as negative controls. After incubation for 5 days at room temperature in the dark without shaking, the spores in each cell were diluted into 5 ml distilled water and the relative fungal growth inhibition was estimated by observing the absorbance at 595 nm.

### Transient expression and *in vitro* antifungal activity analysis of VpPR-10.1 and its mutants in grapevine leaves

An estrogen-inducible ectopic gene expression vector, pER8, was described by Zuo *et al.* [71]. This system shows efficient induction with no toxic effects in transgenic plants. The ORFs of *VpPR-10.1*, *K55N*, *E149G*, and *Y151H* were cloned separately into the plant expression vector pER8 and introduced into *Agrobacterium* strain LBA4404 using electroporation. The expression of each gene was driven by estradiol-inducible expression of the reporter gene in the construct.

To check the *in vitro* antifungal activity of *VpPR-10.1* and its mutants, a susceptible *V. vinifera* named ‘Carignane’ was used. The third and fourth fully expanded leaves from 8-week-old *in vitro* grown ‘Carignane’ plants were analyzed by agro-infiltration. The agro-infiltration assays were performed as described previously with modifications by Guan *et al.* [64]. The Agro-infiltrated leaves were inoculated with *E. necator* as described by Santos-Rosa *et al.* [72] at 1 day post Agro-infiltration. Leaves were submerged abaxial face down in plant tissue culture containers (200 mL, 10 cm height ×6 cm diameter) containing 50 mL of the bacterial suspension. The concentration of the bacterial suspension was measured by Nicolet Evolution 300 UV–VIS spectrophotometer (Thermo Electron Corp., Madison, WI, USA), and it was adjusted to OD_600_ = 0.6 with dilution buffer (10 mM MES, pH 5.6, and 10 mM MgCl_2_). The containers were covered with 0.22 µm microfilters and transferred to a water circulating vacuum pump SHZ-DIII (Shanghai, China). The vacuum infiltration was applied at 0.085 MPa vacuum for 30 min, and released slowly. The surplus bacterial liquid on the surface of the leaves was removed by sterile filter paper, and the leaves were then placed adaxial face up with the petiole wrapped with humidified absorbent cotton in a preservative film-sealed tray. The tray was incubated in chamber at 28 °C and a relative humidity of 80% in the dark for 1 day. The leaves were then induced after 24 h by spraying with 50 mM β-estradiol and 0.1% Tween. The agro-infiltrated leaves were then inoculated with *E. necator* [72].

Hyphae were stained within the leaf using trypan blue as follows. Inoculated leaves were collected at 11 days post-inoculation (dpi). Leaves were put in 6-well plates and 2.5 mL of clearing solution A (acetic acid: ethanol  = 1∶3, v/v) was added to each well. The plate was sealed and shaken at low speed overnight. Clearing solution A was removed from the samples and replaced with 2 mL of clearing solution B (acetic acid: ethanol: glycerol  = 1∶5∶1, v/v/v). After shaking at low speed for at least 3 h, clearing solution B was removed. 2 mL of staining solution (0.3 mL 1% trypan blue stock in dH_2_O, 10 mL lactic acid, 10 mL phenol and 10 mL dH_2_O) was added to each well, and the plate was shaken at low speed overnight. The staining solution was removed from all the leaves, which were rinsed with a little sterilized 60% glycerol to remove all liquid. Samples were then examined by bright-field microscopy. The histological assays were repeated three times. Leaves infiltrated with *Agrobacterium* harboring the empty vector pER8 were used as the negative control. Some of the leaves were used to detect the expression of *VpPR-10.1*, *K55N*, *E149G*, and *Y151H* in infiltrated leaves at several gradient days after infiltration using western blotting.

### Protein extraction and western blot analysis

Protein extraction and western blotting were performed as described previously [73]. For protein isolation, 500 mg of inoculated leaves were homogenized in 1 mL extraction buffer (100 mM Hepes, pH 7.5, 5 mM EDTA, 5 mM EGTA, 15 mM DTT, 15 mM NaF, 50 mM β-glycerophosphate, 1 mM phenylmethylsulfonyl fluoride and 10% glycerol) and incubated for 1 h in cold conditions before being subjected to centrifuge at 18,000× g for 30 min. The supernatant was used as total protein. The protein concentration in the extracts was determined by the Bradford method using bovine serum albumin (BSA) as the standard. For western blotting, 10 µg of total protein per sample was separated by 12% SDS-PAGE using 4% and 15% polyacrylamide in the stacking and resolving gels, respectively. Proteins were then electroblotted onto polyvinylidene difluoride (PVDF) membranes. The membrane was blocked in TTBS (100 mM Tris-HCl, pH 7.5, 0.9% (w/v) NaCl, 0.1% (v/v) Tween-20) containing 5% dry milk for 1 h and then incubated at 4 °C for 1 h with anti-VpPR-10 antiserum diluted 1∶1000. The primary antibody was detected with secondary anti-rabbit IgG at room temperature for 1 h, using nitroblue tetrazolium and 5-bromo-4-chloro-3-indolyl phosphate as substrates.

### Evans blue suspension cell assays

Tobacco BY-2 SCCs were treated with different concentrations of recombinant VpPR-10.1 protein (0 µg·mL^−1^, 25 µg·mL^−1^, 50 µg·mL^−1^, 75 µg·mL^−1^ and 100 µg·mL^−1^) for 24 h at 120 rpm at 25 °C in the darks. Treatments of the same concentrations of BSA were used as controls. To further check for cell death, the tobacco BY-2 SCCs were harvested at 0 h, 6 h, 12 h and 24 h after inoculation with100 µg·mL^−1^ VpPR-10.1, its mutants or BSA. Dead cells were quantified by a previously described method [74]. Cells were collected and incubated with 1 ml of 1% aqueous Evans blue for 5 min, and then washed with deionized water until no further blue eluted from the cells. The samples were examined by bright-field microscopy (Olympus BX51+DP70) to detect dead cells (dark blue). Meanwhile, 50% methanol and 1% SDS solution were added and incubated at 50 °C for 30 min, then quantified spectrophotometrically at A_600_.

### DNA fragmentation assays

To analyze the relationship between the DNA degradation and cell death, we extracted tobacco BY-2 SCCs DNA to check for DNA fragmentation. All samples were taken from cells after treatment with VpPR-10.1 protein (100 µg·mL^−1^), BSA (100 µg·mL^−1^) or VpPR-10.1 antibody (100 µL) for 24 h. Genomic DNA was extracted using the CTAB protocol [75]. Cultured cells were ground in liquid nitrogen with extraction buffer (2% CTAB, 1.4 mol·L^−1^ NaCl, 20 mmol·L^−1^ EDTA, 100 mmol·L^−1^Tris-HCL (pH 8.0), 0.2% β-Mercaptoethanol). After incubating at 65 °C for 30 min, an equal volume of chloroform/isoamylalcohol (24∶1 volume) was added and the DNA precipitated with ethanol. The sample was centrifuged (12,000 rpm, 15 min) at 4 °C and the supernatant was discarded. The pellet was washed with 70% ethanol, centrifuged, the supernatants discarded and the pellets dissolved in TE buffer (10 mM Tris- HCl, 1 mM EDTA, pH 7.4). Genomic DNA (each 10 µg) was analyzed on 1% agarose gel and visualized under UV light.

## Results

### Isolation and analysis of the VpPR-10 cDNA from *V. pseudoreticulata*

**Figure 1:**
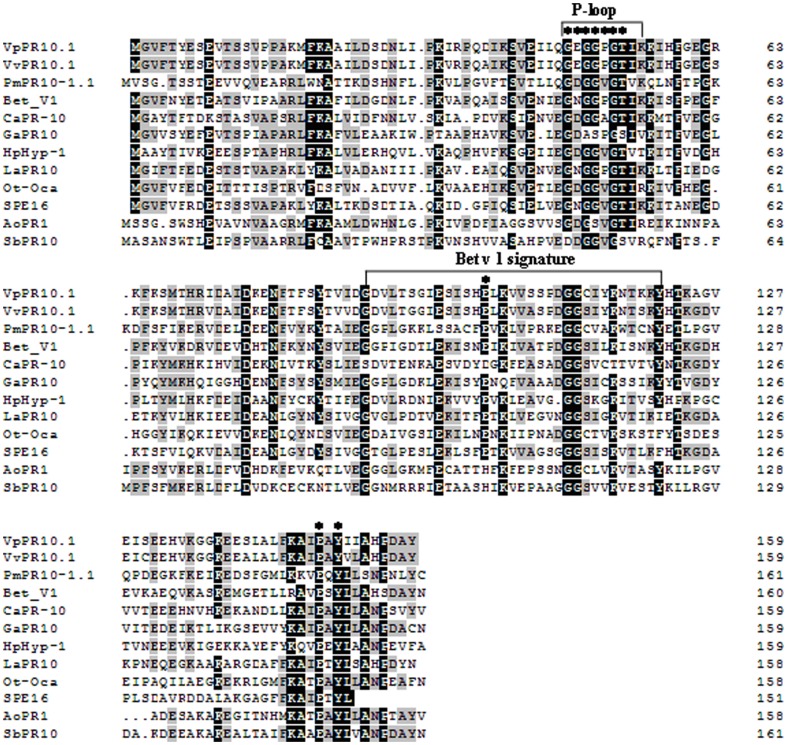
Alignment of the amino acid sequences of VpPR-10.1 and other PR-10 proteins from different plants. The plant sources and GenBank accession numbers of the sequences are shown as follows: *Vitis pseudoreticulata* VpPR10.1 (DQ336289), *Vitis vinifera* VvPR10.1 (AJ291705), *Pinus monticola* PmPR10-1 (AY064193), *Betula verrucosa* Bet V1 (Z72429), *Capsicum annuum* CaPR-10 (AF244121), *Gossypium arboreum* GaPR10 (AF416652), *Hypericum perforatum* HpHyp-1 (AAN65449), *Lupinus albus* LaPR10 (AJ000108), *Oxalis tuberosa* Ot-Oca (AF333436), *Pachyrhizus erosus* SPE16 (AY433943), *Asparagus officinalis* AoPR1 (Q05736) and *Sorghum bicolor* PR10 (U60764). Asterisks indicate strictly conserved amino acid residues of the PR-10 family.

*VpPR-10.1* was isolated from a *V. pseudoreticulata* cDNA library, which was treated with *E. necator*. The clone contains an insert with a complete open reading frame (ORF) of 480 bp, which encodes a peptide of 159 amino acids. The protein has a predicted molecular mass of 17.46 kDa and an isoelectric point of 4.95. The protein was likely to be cytoplasmic, as no signal peptide sequence was detected [61]. The predicted protein has up to 89% amino acid sequence homology with the PR10.1 protein from *V. vinifera* Ugni Blanc. Thus, this clone represented a *PR10.1* gene identified from *V. pseudoreticulata* [61]. The deduced amino acid sequence of *VpPR-10.1* has a conserved P-loop motif GXGGXGXXK and a Betv1 domain, characteristic of many PR-10 proteins (Fig. 1). DNAMAN5.2 software was used to align the predicted amino acid sequence of *VpPR10.1* with several reported PR10 genes containing a P-loop motif and Betv1 domain. Fig. 1 shows that a number of conserved amino acid residues are also found in *VpPR-10.1*.

### Analysis of expressed recombinant VpPR-10.1 proteins

**Figure 2:**
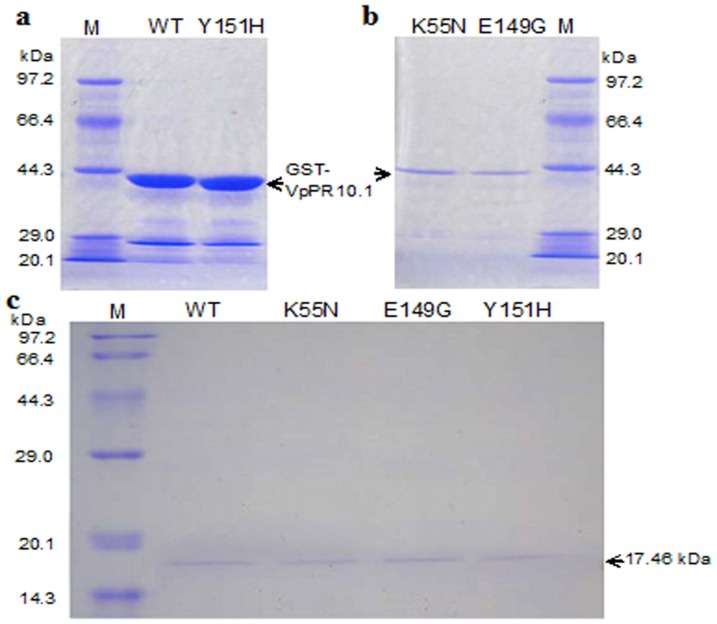
SDS–PAGE analysis of recombinant VpPR10.1 protein and its mutants expressed in *E. Coli*. (a) SDS-PAGE analysis of the purified recombinant VpPR-10.1 protein. Lane 1, protein marker; lane 2, purified wild-type recombinant VpPR-10.1 protein; lane 3, purified recombinant Y151H mutant protein. (b) Lane 1, purified recombinant K55N mutant protein; lane 2, purified recombinant E149G mutant protein; lane 3, protein marker. *VpPR10.1* and its mutant constructs in *E. coli* BL21 (DE3) were induced with 0.1 mM IPTG at 37 °C for 4 h, the gel was stained with Coomassie Blue R-250. (c) SDS-PAGE analysis of the VpPR-10.1 protein without GST. Lane 1, protein marker; lane 2, VpPR-10.1 protein without GST; lane 3, K55N protein without GST; lane 4, E149G protein without GST; lane 5, Y151H protein without GST. The VpPR-10.1 protein product, after GST digestion, was estimated to be approximately 17 kDa.

DNA sequencing was used to proof the wild-type *VpPR-10.1* and to confirm the site-directed mutagenesis of its mutants cloned in pGEX-4T-1 vector. The expression of the wild-type recombinant VpPR-10.1 and its three mutant proteins (K55N, E149G and Y151H) in *E. coli* BL21 (DE3) strain produced a fusion product with a GST tag as a part of the leader sequence of the N-terminus of the protein, which was evident from SDS-PAGE analysis (Fig. 2a). The putative wild-type recombinant VpPR-10.1 and its mutants showed an apparent molecular weight of about 43 kDa, which agrees with the deduced molecular weight from the amino acid sequence (Fig. 2a). For further investigation of nuclease and antifungal activities, the GST tag was removed from the above proteins. Expression of VpPR-10.1 and its mutants without GST in *E. coli* produced a protein of about 17 kDa on SDS-PAGE (Fig. 2b), which approximated to the calculated size of the protein. The purified recombinant proteins were used to conduct all subsequent studies, unless otherwise stated.

### Ribonuclease activity of VpPR-10.1 and its mutant proteins

**Figure 3:**
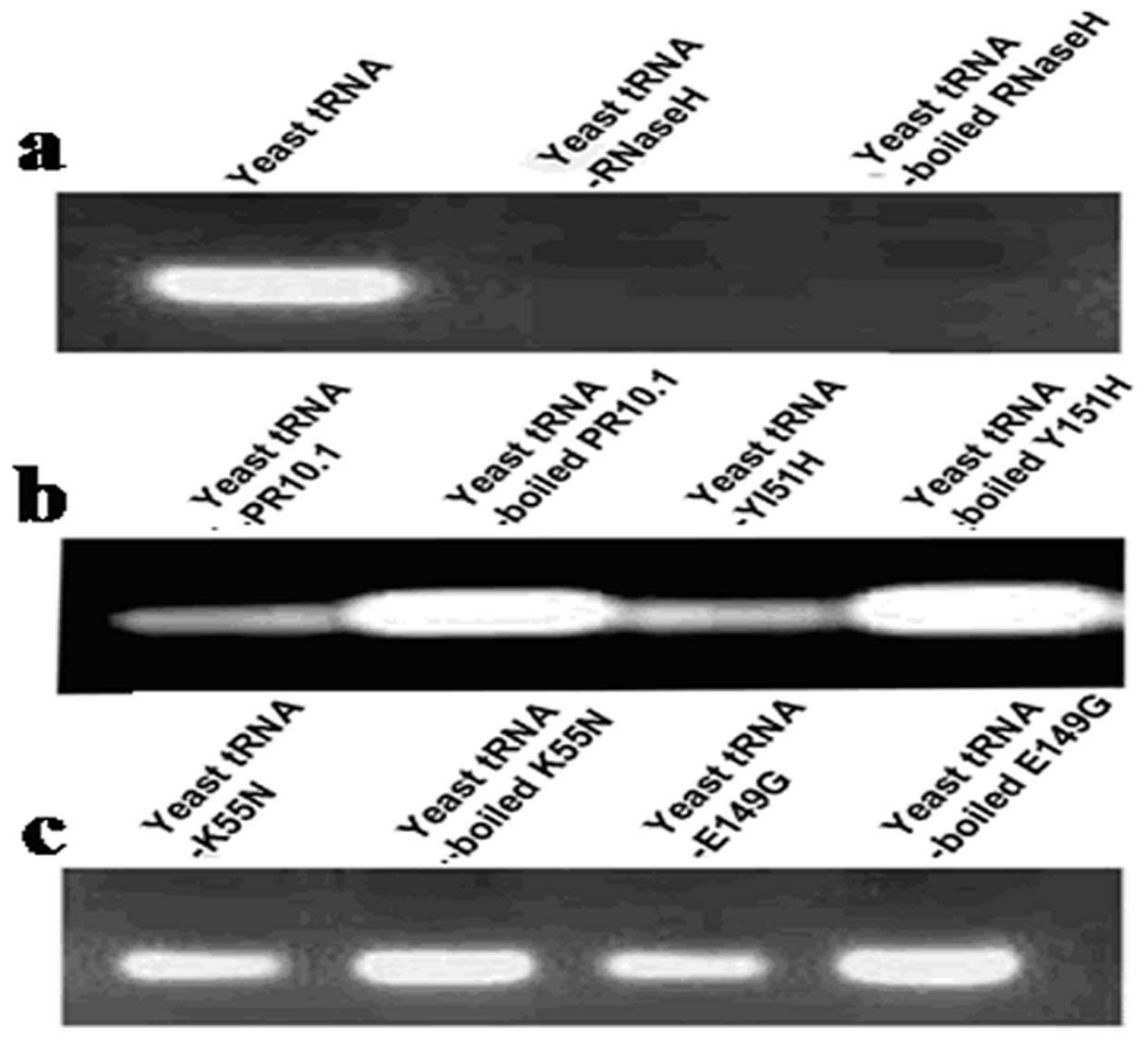
RNase activity assay of purified recombinant VpPR-10.1 and its mutants on yeast total RNA. Samples with each recombinant VpPR-10.1proteins and yeast total RNA in the presence of RNasin were incubated at 37 °C for 30 min. (a) yeast total RNA was usedas the negative control; Because ***RNase H*** is active at **high** temperatures, RNase H and boiled RNase H from E. coli were used as the positive controls. (b) Proteins VpPR-10.1 and Y151H without GST purified from pGEX-4 T-1 in *E. coli*; boiled proteins were used as negative controls. (c) Proteins K55N and E149G without GST purified from pGEX-4 T-1 in *E. coli*; boiled proteins were used as negative controls.

**Figure 4:**
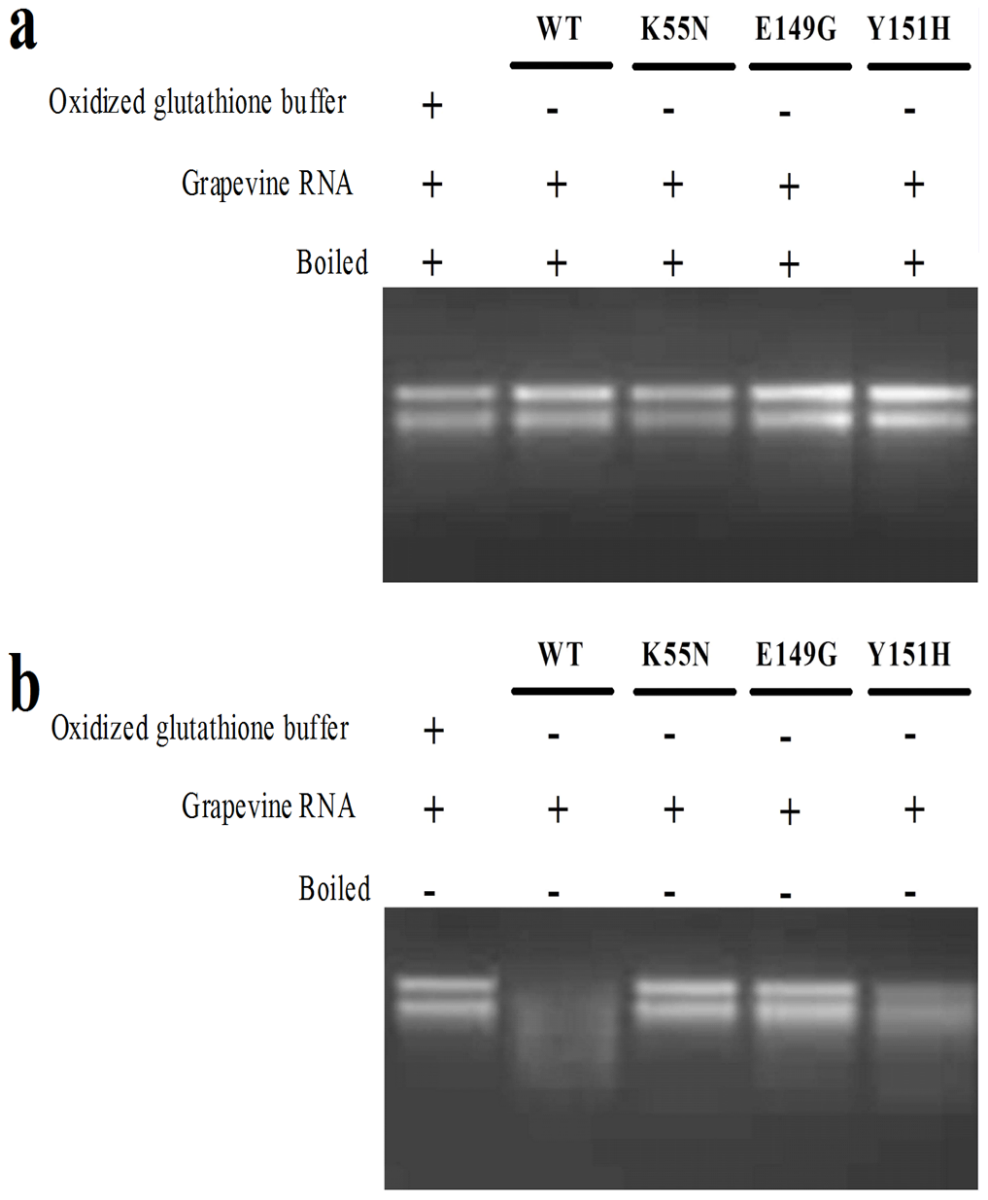
Ribonuclease activities of VpPR-10.1 and mutants assayed on grapevine total RNA. Samples with each recombinant VpPR-10.1 protein and grapevine total RNA in the presence of RNasin were incubated at 37 °C for 30 min. (a) Boiled proteins without GST purified from pGEX-4 T-1 in E. coli and elution buffer in the presence of RNasin were used as negative controls. (b) Recombinant proteins VpPR-10.1, Y151H, K55N, and E149G without GST were incubated with grapevine total RNA in the presence of RNasin. Mutant proteins K55N and E149G lost the function of degrading RNA. Elution buffer was used as a negative control.

According to known three-dimensional structures [76], [77], three amino acids (K55N, E149G and Y151H) were predicted to lie in the active sites because their side chains have functional groups presumably involved in the catalytic reaction. Thus, the wild-type VpPR-10.1, mutants K55N, E149G and Y151H were constructed and their effects on ribonuclease activities were observed. Differential RNase activities of wild-type and mutant VpPR-10.1 proteins were observed in all three RNase assays as shown in Figs. 3 and 4. In the yeast total RNA degradation assay, the recombinant VpPR-10.1 protein showed significant ribonucleolytic activity, where yeast total RNA was almost degraded within 30 min of incubation and was not inhibited by RNase inhibitor (RNasin) (Fig. 3b, lane 1). The negative control with boiled VpPR-10.1 protein was not found to have activity (Fig. 3b, lane 2). Two positive controls, RNase H and boiled RNase H (**RNase H** is active at **high temperature**) from E. coli, degraded the yeast total RNA sample at the time point designated (Fig. 3a, lanes 2 and 3). By contrast, degradation of yeast total RNA was not observed when incubated in elution buffer only (Fig. 3a, lane 1). In the case of mutant Y151H, the RNase activity was also strong and was inactivated by heating (Fig. 3b, lanes 3 and 4), suggesting that conserved amino acid residue Tyr 151 was not critical to the RNase activity. Meanwhile, when incubated with the same amounts of K55N and E149G proteins, the most of yeast total RNA existed (Fig. 3c). These data indicated that VpPR-10.1 protein possesses RNase activity, and amino acids Lys55 and Glu149 are critical for that activity.

Moreover, to better understand the activity of plant RNA, one RNA degradation assay was performed on plant RNA. Total RNA isolated from *V. pseudoreticulata* leaves was incubated with wild-type recombinant protein VpPR-10.1 and its mutants. No degradation of *V. pseudoreticulata* total RNA was observed when it was incubated with elution buffer or any of the boiled proteins (Fig. 4a). On the other hand, K55N and E149G proteins showed no RNase activity, whereas Y151H protein lost a little of its activity compared with the wild-type VpPR-10.1, for which total RNA degradation was clearly visible (Fig. 4b). The results showed similar degradation patterns by VpPR-10.1 and its mutant proteins to those obtained using yeast tRNA.

### DNase activity of VpPR-10.1 and its mutant proteins

**Figure 5:**
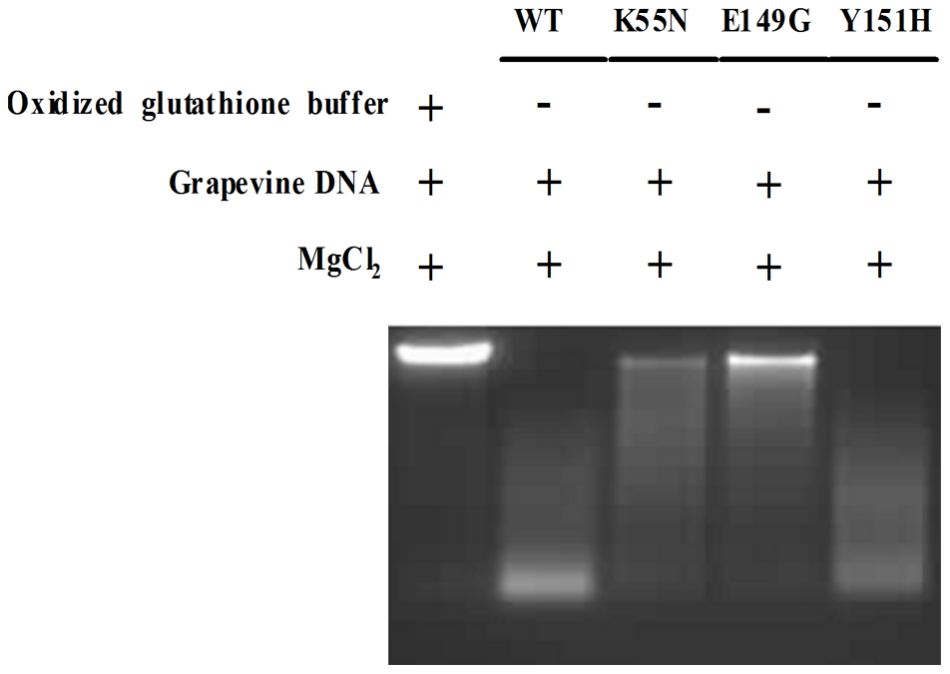
DNase activity of VpPR-10.1 and its mutants assayed on *V. pseudoreticulata* genomic DNA. Samples with each recombinant VpPR-10.1protein and pseudoreticulata genomic DNA were incubated at 37°C for 30 min and then subjected to agarose gel electrophoresis. Comparisom of DNase activities of recombinant VpPR-10.1 proteins was performed in the presence of 2.5 mM MgCl_2_. Elution buffer was used as negative control. All proteins were without GST.

Different DNase activities of wild-type and mutant VpPR-10.1 proteins were observed in the DNase assay using genomic DNA from *V. pseudoreticulata*. As shown in Fig. 5, no degradation of genomic DNA was observed upon incubation with elution buffer (oxidized glutathione buffer) only, which suggested that there was no contamination from the buffer and plant DNA samples. However, when incubated with the wild-type recombinant VpPR-10.1 protein with MgCl_2_, genomic DNA degradation was clearly visible. These data indicated that VpPR-10.1 protein possesses DNase activity.

To investigate the functional importance of the conserved amino acid residues related to DNase activity, we used the three VpPR-10.1 mutants (K55N, E149G, Y151H). After digestion, K55N and E149G displayed significantly lower DNase activities than the wild-type (Fig. 5). Although they did not completely abolish the DNase activity of VpPR-10.1, they decreased most of the activity; hence, these two conserved amino acid residues are involved in the DNase activity of VpPR-10.1. In contrast, Y151H retained almost all its DNase activity compared with wild-type VpPR-10.1 (Fig. 5). Our results showed that the purified wild-type recombinant protein VpPR-10.1 had DNase activity and the effect of VpPR-10.1 on the degradation of DNA was associated with two conserved amino acid residues (Lys55 and Gly149), but not with Tyr151.

### Antifungal activity of VpPR-10.1

**Figure 6:**
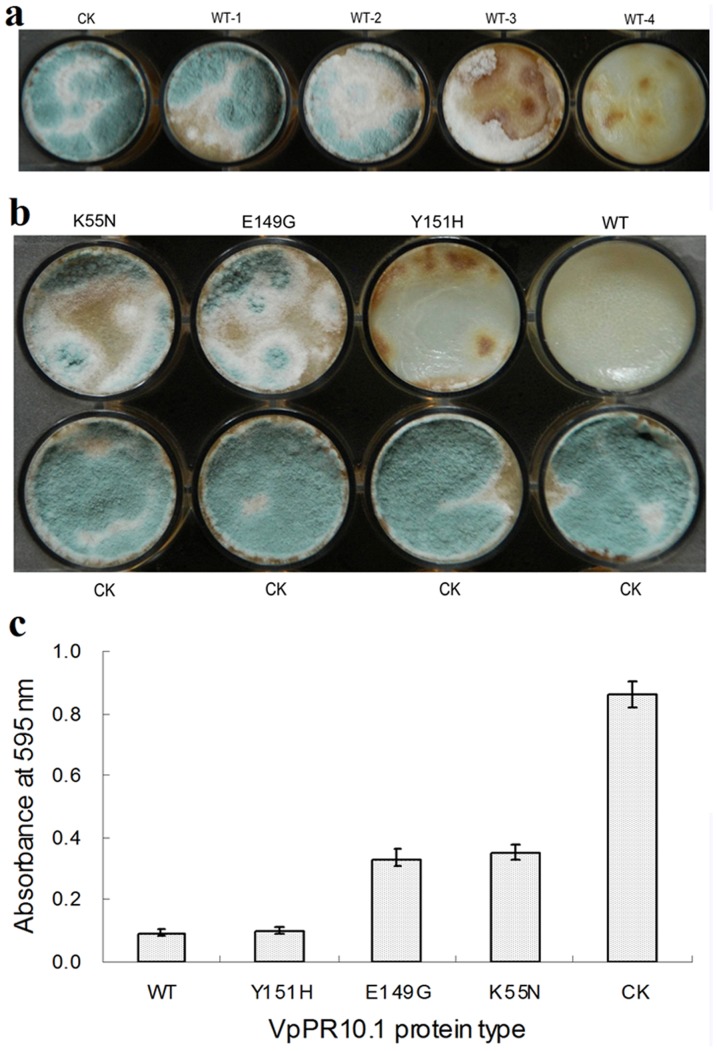
Antifungal activity assays of VpPR-10.1 toward *A. alternate*. (a) *A. alternate* was grown on PDB medium in the presence of purified wild-type recombinant VpPR-10.1 and evaluated after incubating for 5 days at room temperature. CK, oxidized glutathione buffer (the protein elution buffer) was used as qa negative control; WT-1, 20 µg of VpPR-10.1; WT-2, 40 µg of VpPR-10.1; WT-3, 60 µg of VpPR-10.1; WT-4, 80 µg of VpPR-10.1. (b) Analysis of *A. alternate* grown on PDB medium in the presence of purified wild-type recombinant VpPR-10.1 and mutant proteins at 80 µg·mL^−1^. (c) *A. alternate* grown on PDB medium in the presence of 80 µg·mL^−1^ purified wild-type recombinant VpPR-10.1 and mutant proteins were collected and diluted into 5 ml distilled water, then estimated by observing the absorbance at 595 nm. Each point on the plot is the average of three independent determinations.

Different concentrations of VpPR-10.1 showed distinctive inhibition of *Alternaria alternata f.* sp. Lycopersici (Fig. 6a). In assays of VpPR-10.1 protein against *A. alternata*, concentrations of 60 and 80 µg·mL^−1^ were found to effectively inhibit fungal growth (Fig. 6a). Thus, for antifungal activity analysis of wild-type VpPR-10.1 and its mutants, a concentration of 80 µg·mL^−1^ of each protein was used. The results showed that Y151H protein retained almost all its antifungal activity and inhibited growth of *A. alternate* significantly at the designated concentration. K55N and E149G proteins showed quite less level of inhibitory effect on pathogen growth, indicating that both had lost nearly all their activities compared with the wild-type (Fig. 6b). As negative controls, using oxidized glutathione buffer (the protein elution buffer) alone was not observed (CK) (Fig. 6b). Also, similar results were obtained when the spores from each sample of treated cells were diluted into 5 mL distilled water and estimated by observing the absorbance at 595 nm (Fig. 6c).

### Over-expression and the *in vivo* antifungal activities of VpPR-10.1 gene in grapevine leaves

**Figure 7:**
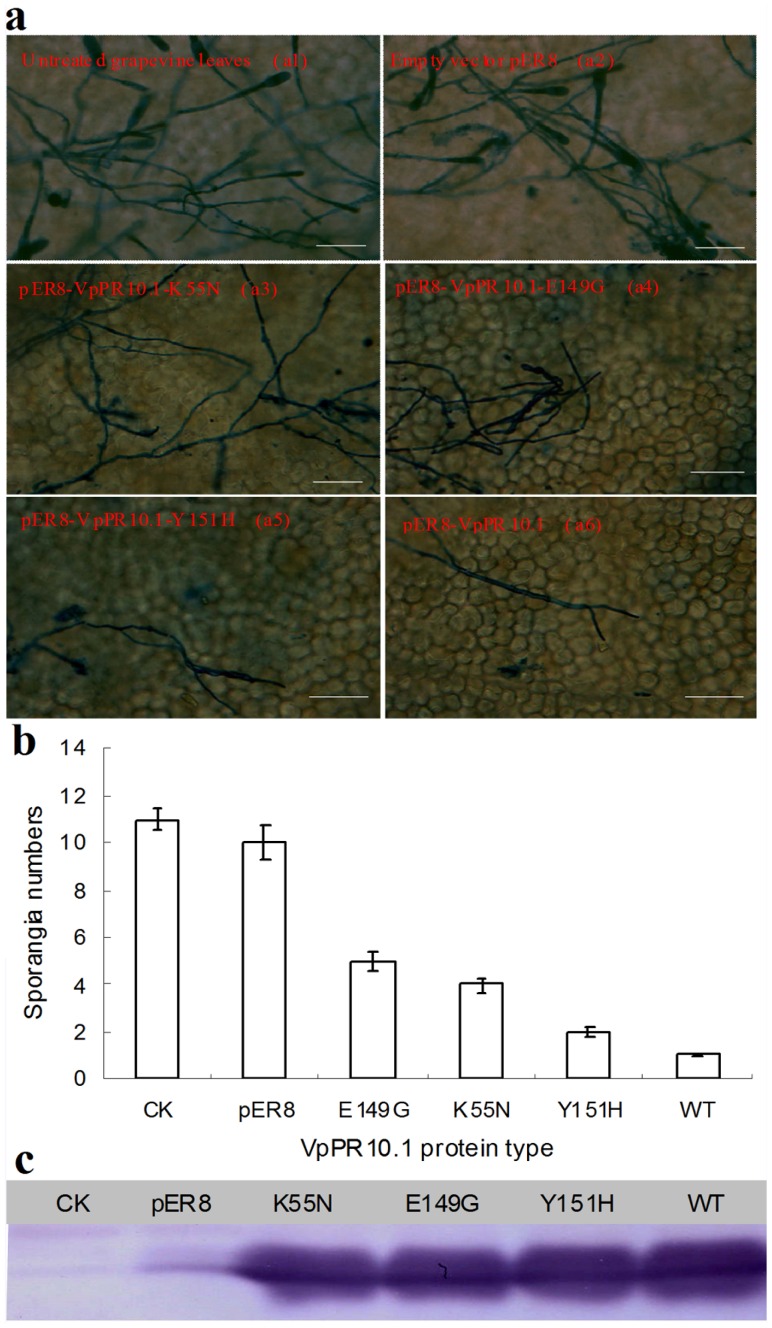
Transient expression and anti-fungal activity assay of VpPR-10.1 against *E. necator* in grapevine leaves. (a)Trypan blue staining of infiltrated grapevine leaves were collected at 11 days post-inoculation (dpi) with *E. necator*. Pictures were representative of six independent experiments and ten leaves per experimental condition. They were grapevine leaves that were infiltrated with *Agrobacterium* harboring the empty vector pER8, *K55N*, *E149G*, *Y151H*, and *VpPR-10.1*, respectively. Untreated grapevine leaves were used as negative control. Sporangia and hyphae were shown as blue spots and lines, respectively. Scale bar = 10 µm.(b) Sporangia numbers grown on the untreated grapevine leaves and the leaves that infiltrated with *Agrobacterium* harboring the empty vector pER8, *VpPR-10.1*and mutants. (c) Western blot analysis of VpPR-10.1 and mutant proteins in leaves inoculated with *E. necator*. Soluble proteins were separated by SDS–PAGE, blotted onto a PVDF membrane and reacted with antiserum against VpPR-10.1.

The leaves from ‘Carignane’ were infiltrated with *Agrobacterium* harboring each of five different constructs: pER8-*VpPR.10.1*, pER8-*K55N*, pER8-*E149G*, pER8-*Y151H*, or empty vector pER8. Microscopic images of infiltrated leaves stained with trypan blue and bar graphs of spore numbers are shown Fig. 7. After inoculation, the sporangia of *E. necator* were successfully attached on the leaves' surface. As controls, histological observations of hyphal growth at 11 days post-inoculation revealed the powdery mildew symptoms induced by *E. necator* in the vector-transformed and untransformed leaves (Fig. 7a–7b). Infiltration of the leaves with either wild-type VpPR-10.1 or VpPR-10.1-Y151H significantly reduced the numbers of mycelia and spores compared with the controls (Fig. 7a–7b). Interestingly, VpPR-10.1-K55N- and VpPR-10.1- E149G-infiltrated leaves exhibited quite a less level of protection compared to the wild type (Fig. 7a–7b). To exclude the influence of protein expression levels, we checked the amount of proteins in different infiltrated leaves using western blotting. Similar levels of the various forms of VpPR-10.1 were found in the infiltrated leaves (Fig. 7c). Taken together, these results revealed that over-expression of VpPR10.1 in grapevine leaves could enhance host resistance against *E. necator*, which crucially depends on Lys55 and Glu149.

### Recombinant VpPR-10.1 causes cell death in tobacco suspension-cultured cells (SCCs)

**Figure 8:**
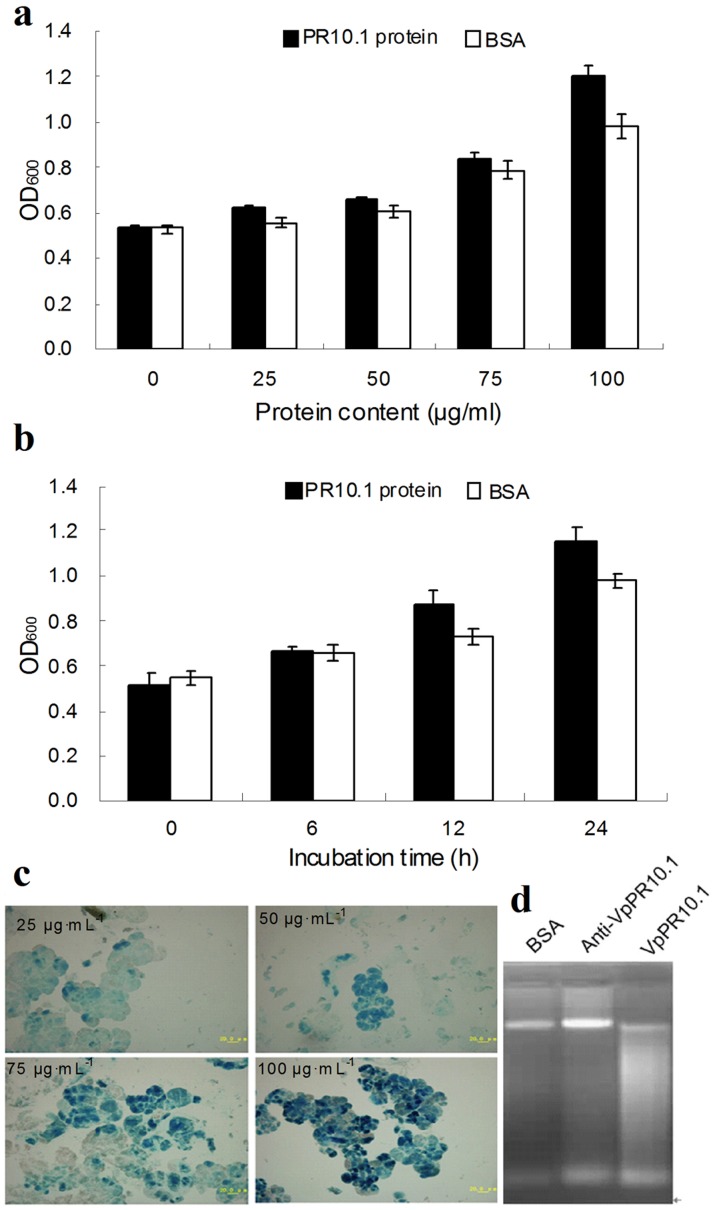
Recombinant VpPR-10.1 protein causes cell death of tobacco BY-2 cells. (a) Effect of different concentration of VpPR-10.1 protein (25, 50, 75 and 100 µg·mL^−1^) on cell death. BSA (100 µg·mL^−1^) was used as a negative control. (b) Prolonged incubation (0, 6, 12 and 24 h) of cultured cells with VpPR-10.1 protein (100 µg·mL^−1^) showed difference on cell death. (c) Morphology of cells after incubating with different concentration of VpPR-10.1 protein for 24 h (25 µg·mL^−1^, 50 µg·mL^−1^, 75 µg·mL^−1^, 100 µg·mL^−1^). Blue color indicated cell death. (d) DNA fragmentation analyses of tobacco BY-2 cells treated with recombinant VpPR-10.1 protein. Lane 1, tobacco BY-2 cells were treated with BSA (control; 100 µg·mL^−1^); lane 2, tobacco BY-2 cells were treated with 100 µg·mL^−1^ of VpPR-10.1 protein and 100 µL antibody; lane 3, tobacco BY-2 cells were treated with 100 µg·mL^−1^ of VpPR-10.1 protein.

We investigated the effect of recombinant VpPR-10.1 on plant cells. Tobacco BY-2 SCCs were co-incubated with different concentrations of the VpPR-10.1 protein and BSA as a control (Fig. 8). When incubated with increasing concentrations of the VpPR-10.1 protein, increasing amounts of cell death were observed (Fig. 8a). A time course experiment was performed. As shown in Fig. 8b, 100 µg·mL^−1^ of VpPR-10.1 specifically induced a strong increase in cell death compared with the control cells, after 12 h of treatment. Thus, induction of cell death mediated by VpPR-10.1 was also dependent on treatment time. Sensitivity of SCCs to VpPR-10.1 was determined by staining the treated cells with Evans blue. These results showed that SCCs treated with increasing concentrations of the protein turned a darker blue color (*i.e.* more cell death) (Fig. 8c). Cells treated with 25 µg·mL^−1^ of VpPR-10.1 remained light blue, whereas 50 µg·mL^−1^ of VpPR-10.1 caused obvious cell death, indicating that at this level VpPR-10.1 is sufficient to induce cell death in tobacco SCCs (Fig. 8c).

To analyze whether VpPR-10.1-induced cell death is associated with DNA degradation, we extracted tobacco BY-2 SCCs DNA after treatment with BSA (100 µg·mL^−1^), VpPR-10.1 antibody (100 µL), and VpPR-10 (100 µg·mL^−1^) for 24 h. DNA fragmentation analysis revealed that VpPR-10-treated cells showed specific DNA degradation, implying a strong relationship between VpPR-10-mediated DNA degradation and cell death in tobacco SCCs.

## Discussion

Pathogenesis-related proteins of the PR10 family are believed to have a role in plant defense [4]. The reported 17 grapevine PR10 related genes have high sequence similarity [60], but they display different basal expression levels in healthy leaves and show different responses to pathogen attacks. Moreover, in the fungal-resistant grapevine *V. pseudoreticulata*, the transcripts of *PR-10.1*, *PR-10.2*, *PR-10.3* and *PR-10.7* were detectable in non-treated leaves [60]. Only *VvPR-10.1* was up-regulated during a pathogen interaction with *Pseudomonas syringae* in the cultivar Ugni Blanc [34]. Thus, it seems that only a few members of the grapevine PR10 gene family are involved in the response to pathogen infection in leaves. Fig. 1 shows the sequence alignment of *VpPR10.1* and other PR10 class proteins. Comparison of the amino acid sequence reveals that *VpPR10.1* has both P-loop and Bet v 1 motifs characteristic of other reported PRs, and they share several conserved amino acids at positions in the P-loop and Bet v 1 motifs. In other PR10s having RNase activity, Tyr148 and Glu150 are conserved; however, in VpPR-10.1 these are replaced by Glu149 and Tyr151, respectively. However, Lys54 is also conserved in *AhPR10*. The definitive biological function of VpPR-10.1 has not been fully determined. However, sequence similarity among these PR10 proteins suggests that they may have nuclease activity. Our mutational strategy involved mutants VpPR10.1-K55N, VpPR10.1-E149G and VpPR10.1-Y151H. These amino acids were chosen because they were either in the region corresponding to the P-loop (Lys55), or are proposed to be involved in the catalytic reaction (Glu149, and Tyr151). In this study, we have constructed these three *VpPR10.1* mutants from the wild-type protein from pathogen-induced *V. pseudoreticulata* leaves.

Recently, PR-10 proteins in other plants have been shown to possess RNase activity, such as GaPR-10 from *Gossypium arboreum* [78], SPE-16, a PR-10 protein from *Pachyrrhizus erosus* [24], CaPR-10 from hot pepper (*Capsicum annuum*) [31] and Bet v 1 from birch (*Betula verrucosa*) pollens [19]. In this study, the purified recombinant VpPR10.1 showed RNase activity in both RNase assays (Figs. 3 and 4) as predicted by its amino acid sequence. The P-loop (GxGGxGxxK), a motif believed to be involved in ATP or GTP binding, has been considered to be the possible RNA phosphate-binding site associated with the ribonucleotlytic activity [10], [52]. The P-loop has been shown to be critical for the RNase activity in SPE-16 [24]. Reduction of RNase activity of AhPR10.1-K54N suggested the involvement of Lys54 in its RNase activity [56]. Here, Lys 55 also appears to play a more crucial role in the RNase activity of VpPR-10.1 because this mutant causes almost complete loss of its RNase activity (Figs. 3 and 4). A similar role of Lys55 in GaPR10 (*Gossypium arboreum*) in RNase activity was reported [24], [78]. In addition, our data provided experimental evidence to indicate an essential role of the carboxyl group of Glu149 for catalysis. By contrast, Tyr151 is not as important as these two amino acid residues, at least for VpPR-10.1. It seems that the P-loop motif and amino acid residue Glu149 play a major role in ribonucleic acid degradation.

Although many studies have shown RNase activity in the PR-10 proteins, little is known about DNA degradation by VpPR-10.1. Previously, Kim *et al.* [62] proposed a role for PBZ1 in cell death that was supported by DNA fragmentation analysis. Thus, we detected the DNase activity of VpPR-10.1 against grapevine total DNA (Fig. 5). As with the same pattern of RNase activity, mutants K55N and E149G lost their DNase activities, whereas VpPR-10.1 and VpPR10.1-Y151H showed DNase activities against the host genomic DNA. It appears that the RNase and DNase functions have been retained during evolution of plant PR-10 proteins. The ribonuclease activity of VpPR-10.1 proteins is related to their fungicidal properties. According reports previously, the RNase activity may be important both for direct impact (due to the destruction of the mRNA pool of fungi at the penetration of nuclease molecules into the cells of the pathogen) and for the induction of apoptosis of plant cells at the site of infestation (hypersensitivity reaction) [79].

At the same time, direct evidence of antifungal activity conferred by PR-10 proteins comes only from *in vitro* microbe inhibition experiments. For example, the recombinant CaPR-10 protein from hot pepper (*Capsicum annuum*) inhibited the growth of the oomycete pathogen *P. capsici* [31]. SsPR10 from *Solanum surattense* shows both ribonucleolytic and antimicrobial activity [55], but the results of over-expressing PR-10 genes in transgenic plants were not the same. Unlike many defense-related genes described in similar systems, expression of PR-10-homologous SRG1-like genes does not correlate with resistance to *Colletotrichum trifolii* [80]. Similar negative results have also been observed in the studies of pea PR-10.1 [81]. All data indicated the possible selectivity of inhibition by the protein. In the present study, VpPR10.1 protein showed strong growth inhibition against *A. alternate* and over-expression of *VpPR10.1* in *V. vinifera* enhanced resistance to *E. necator* (Figs. 6 and 7). AhPR10 appears to exert its antifungal activity upon entering into the fungal hyphae of sensitive fungi, as the protein is not internalized in *S. roxsii* [56]. Similar observations were also made for antifungal histatins against *C. albicans* [82], [83]. The non-inhibition of the growth of *A. alternate* and *E. necator* by VpPR10.1-K55N and VpPR10.1-E149G, which lack RNase and DNase activity, suggested the possible role of the RNase and DNase functions in fungal inhibition. The AhPR10-K54N protein also showed no inhibition of the growth of *F. oxysporum* and *R. solani* [56]. However, the Y151H mutant protein, which retained its RNase and DNase activities, showed strong antifungal activity. Taken together, the results implied that the antifungal activities of *VpPR10.1* have a great influence on resistance to *E. necator* in host plants and that two conserved amino acid residues, Lys55 and Glu149, are involved in this activity.

Programmed cell death (PCD) is a hallmark of PR10 proteins; therefore, we monitored cell death of tobacco BY-2 SCCs treated with VpPR10.1 protein for different concentrations and times (Fig. 8). The results showed induction of cell death when treated with 100 µg·mL^−1^ of VpPR10.1 protein (Fig. 8a and c), and that this was significant after 12 h (Fig. 8b). The assay was also applied to independently determine VpPR10.1-induced genomic DNA fragmentation in tobacco BY-2SCCs. Treatment with VpPR10.1 caused positive signals of DNA fragmentation upon electrophoretic analysis (Fig. 8d). Previously, in plant cells, DNA fragmentation was documented in tobacco BY-2 cells undergoing PCD in response to abiotic stress [84]. PBZ1, a PR-10-like protein with *in vitro* RNase activity, caused DNA fragmentation in rice, which is a recognized sign of PCD in plants [62]. Thus, the results show that VpPR10.1 causes programmed cell death in tobacco BY-2 cells.

Increased ribonuclease activity has been observed in tobacco leaves during the hypersensitive response to tobacco mosaic virus [85]. It was reported that a PBZ1 protein with DNase activity was related to plant defense [62]. Meanwhile, Chadha and Das [56] reported FITC-labeled AhPR10 had lost its ribonuclease activity did not inhibit fungal growth. Our study detected the DNase activity of VpPR-10.1 and demonstrated DNA fragmentation in tobacco suspension-cultured cells incubating with the VpPR-10.1 proteins, which showed mutants that lacking nuclease activities had no antifungal activities. Thus, the DNase activity of PR10 proteins might also play a significant role in PCD in plants. The observed loss of antifungal activities in grapevine PR-10.1 mutant proteins lacking RNase and DNase activities suggests an important protective role of VpPR-10 by degrading DNA or RNA of either foreign, invading pathogens or the host.

Clearly, more studies will do to completely understand the role of *VpPR-10.1* in the defense mechanism. Screening for proteins that interact with VpPR10.1 in Chinese wild *V. pseudoreticulata* ‘Baihe-35-1’ cDNA library using the yeast-two hybrid system, we have already identified several VpPR10.1 partner proteins that are associated with defensive against pathogens and abiotic stresses, such as Trx h2, Grx C9, and GLOX [86]. Further analyses of these genes should help in determining VpPR10.1's function and in identifying new components of the PCD pathway in grapevine and other plants.

## Conclusions

VpPR10.1, isolated from fungal-resistant *V. pseudoreticulata*, shares conserved features with other PR-10 genes. The recombinant Vp-PR10.1 protein showed DNase and RNase activities and inhibited the growth of the fungus *A. alternate*. Over-expression of *VpPR-10.1* in susceptible *V. vinifera* leaves enhanced the host resistance to *E. necator*. The study of conserved amino acid residues revealed a critical involvement of Lys55 and Glu149, but not Tyr151, in VpPR10.1's activities. Combined with the results from the assays of antifungal activities, we propose that the RNase and DNase activities of VpPR10.1 likely constitute the biochemical basis for its defensive function. Obvious DNA fragmentation in plant cells treated with VpPR10.1 represents a recognized signal for PCD. Collectively, these results suggest that the VpPR10.1 protein plays a dual role in host defense against fungal infection in grapes.
